# What radiologists should know about tomographic evaluation of acute diverticulitis of the colon

**DOI:** 10.1590/0100-3984.2015.0227

**Published:** 2017

**Authors:** Aline de Araújo Naves, Giuseppe D'Ippolito, Luis Ronan Marquez Ferreira Souza, Sílvia Portela Borges, Glênio Moraes Fernandes

**Affiliations:** 1 Full Member of the Colégio Brasileiro de Radiologia e Diagnóstico por Imagem (CBR), Specialist Student at the Center for Imaging Sciences and Medical Physics of the Hospital das Clínicas da Faculdade de Medicina de Ribeirão Preto da Universidade de São Paulo (HCFMRP-USP), Ribeirão Preto, SP, Brazil; 2 Tenured Professor in the Department of Diagnostic Imaging of the Escola Paulista de Medicina da Universidade Federal de São Paulo (EPM/Unifesp), São Paulo, SP, Brazil; 3 PhD, Associate Professor of Radiology at the Universidade Federal do Triângulo Mineiro (UFTM), Uberaba, MG, Brazil; 4 Medical Student at the Universidade Federal do Triângulo Mineiro (UFTM), Uberaba, MG, Brazil; 5 Specialist in Coloproctology, Preceptor at the Universidade Federal do Triângulo Mineiro (UFTM), Uberaba, MG, Brazil

**Keywords:** Diverticulitis, colonic, Abdomen, acute, Tomography, X-ray computed

## Abstract

Acute diverticulitis of the colon is a common indication for computed tomography, and its diagnosis and complications are essential to determining the proper treatment and establishing the prognosis. The adaptation of the surgical classification for computed tomography has allowed the extent of intestinal inflammation to be established, the computed tomography findings correlating with the indication for treatment. In addition, computed tomography has proven able to distinguish among the main differential diagnoses of diverticulitis. This pictorial essay aims to present the computed tomography technique, main radiological signs, major complications, and differential diagnoses, as well as to review the classification of acute diverticulitis.

## Abstract (pt)

A diverticulite aguda dos cólons é uma indicação frequente de exame tomográfico, sendo o seu diagnóstico e das suas complicações fundamental para determinar uma adequada conduta terapêutica e estabelecer o prognóstico. A adaptação da classificação cirúrgica para a tomografia computadorizada permitiu estabelecer a extensão do processo inflamatório intestinal, correlacionando o quadro tomográfico com a indicação de tratamento. Além disto, a tomografia computadorizada tem demonstrado ser capaz de distinguir os principais diagnósticos diferenciais da diverticulite aguda dos cólons. Este ensaio iconográfico tem por objetivo apresentar a técnica de exame tomográfico, os principais sinais radiológicos, e revisar a classificação e as principias complicações e diagnósticos diferenciais da diverticulite aguda dos cólons.

## INTRODUCTION

Diverticula are small sacs of mucosa and submucosa that protrude through the muscle layer of the wall of the intestinal loop, between the taenia coli and the mesentery, at the point of penetration of the blood vessel. Acute colonic diverticulitis (ACD) is the most common complication of diverticular disease, and it is estimated that up to 25% of ACD patients will present acute inflammatory abdomen during the course of their lives^(1)^.

Imaging tests play a crucial role in the appropriate management of ACD. Among such tests, computed tomography (CT) is considered the method of choice in the protocols established by the American Society of Colon and Rectal Surgeons^(2)^, because it allows rapid diagnosis and has an accuracy of over 90%^(1)^.

This pictorial essay aims to present the CT examination technique and the main radiological signs of ACD. We also review its classification, main complications, and differential diagnoses.

## CT TECHNIQUES

For the CT evaluation of patients with suspected ACD, certain protocol options can be adopted depending on the clinical condition of each patient, and the contrast agent can be administered via the intravenous, rectal, or oral route. It is recommended that the image acquisition extend from the diaphragm to the pubic symphysis^(3)^. The technical parameters kV and mAs should be adjusted depending on the waist circumference of the patient, in order to optimize the image quality and radiation dose^(3)^.

The contrast enhancement of the colonic loops facilitates the detection of ACD and its complications, such as perforation, fistulas, and abscesses. To visualize the entire colon, 500–1000 mL of iodinated contrast, diluted 5–10%, should be administered rectally, without pressure, the patient being rotated in order to advance the contrast up to the cecum^(4)^. The introduction of air and water into the rectum does not interfere with CT colonoscopy or CT angiography.

Intravenous iodinated contrast medium facilitates the evaluation of the extracolonic extent of ACD and can be used at a dose of 2 mL/kg, delivered at a velocity of 2.5–3.0 mL/s. Images can be acquired at 60–90 s after initiation of the contrast administration^(4)^.

The use of the oral contrast agent is less frequent in the literature and in daily practice, due to the long preparation time and the large volume to be ingested^(4)^.

## TOMOGRAPHIC ASPECTS

The CT diagnosis of ACD is made on the basis of the following findings:

– Diverticulitis (Figure 1), which has a sensitivity of 43% and a specificity of 100%^(5)^.
  **Figure 1:**
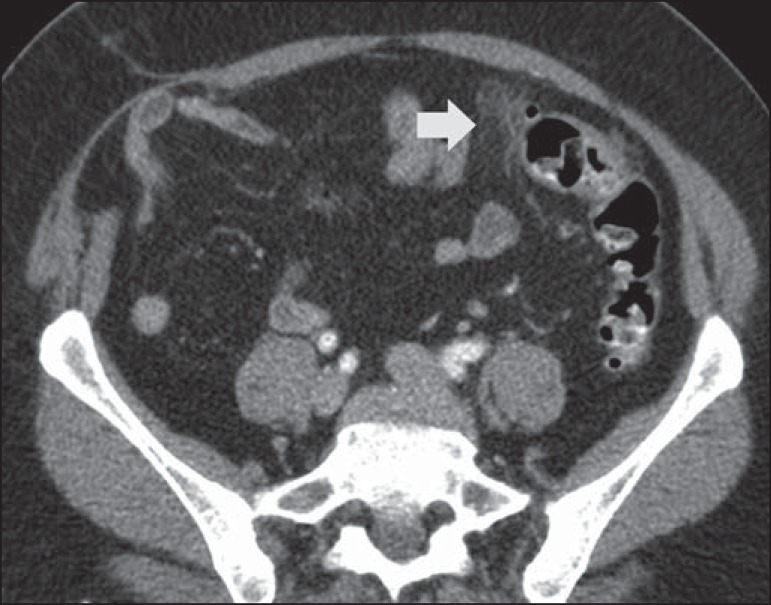
Inflamed diverticulum. Intravenous contrast-enhanced axial CT of the abdomen, showing the diverticulum with discrete wall thickening (arrow) and increased attenuation of pericolonic fat.
– Intestinal wall thickening (Figure 2), which has a sensitivity of 96% and a specificity of 91%^(5)^.
  **Figure 2:**
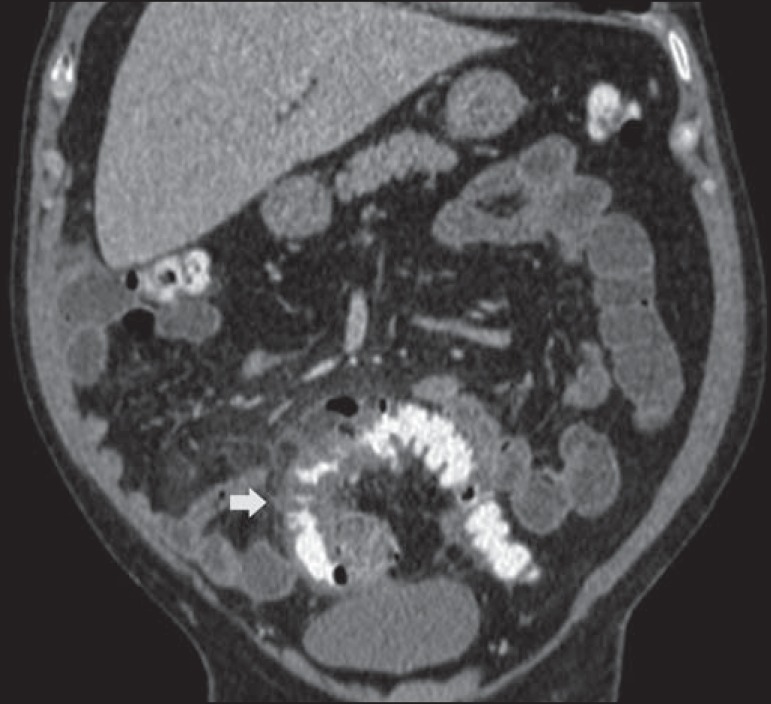
Wall thickening. Intravenous and rectal contrast-enhanced coronal CT scan of the abdomen, showing colonic diverticula associated with thickening of the intestinal wall to > 1.0 cm, with an extent of 8.0 cm (arrow).
– Signs of inflammation in the pericolonic fat and thickening of the lateroconal fascia (Figure 3), which have a sensitivity of 95% and 50%, respectively, and a specificity of 90% and 100%, respectively^(5)^.
  **Figure 3:**
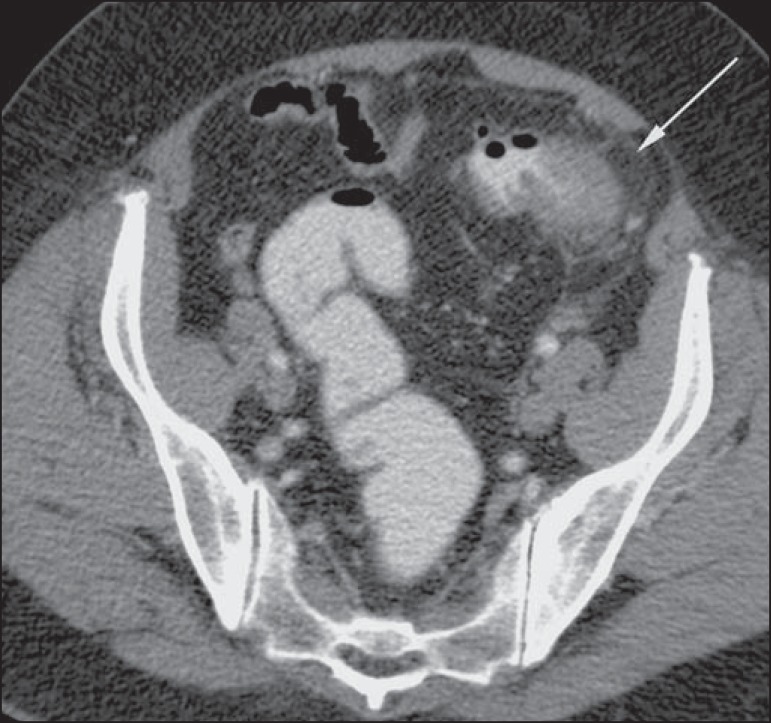
Inflammatory signs in pericolonic fat. Intravenous and rectal contrastenhanced axillary CT, in the axial plane, showing increased mesenteric fat attenuation (arrow) adjacent to the inflammatory process in the diverticula.
– Signs of intestinal perforation (Figure 4), which have a sensitivity of 30% and a specificity of 100%^(5)^.
  **Figure 4:**
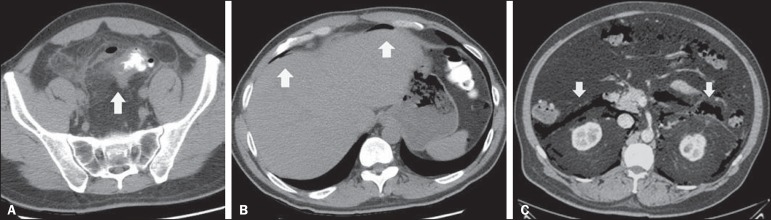
**A,B:** Rectal contrast-enhanced CT of abdomen showing signs of acute diverticulitis, characterized by sigmoid wall thickening, diverticula, and increased regional fat density (arrow in **A**), as well as extraluminal gas, indicating pneumoperitoneum (arrows in **B**). **C**: Signs of intestinal perforation. Intravenous contrast-enhanced abdominal CT, in the axial plane, showing pneumoretroperitoneum (arrows) secondary to diverticulitis.
– Pericolonic or distant abscess (Figure 5), which has a sensitivity of 58% and a specificity of 99%^(5)^.
  **Figure 5:**
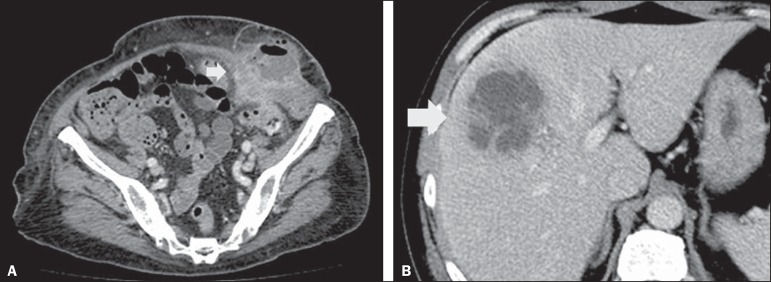
Pericolonic/distal abscess. Intravenous contrast-enhanced abdominal CT, in the axial plane, showing heterogenous fluid collections (arrows) surrounded by a hyperintense halo with contrast enhancement.
– Fistulas with adjacent organs (Figure 6).
  **Figure 6:**
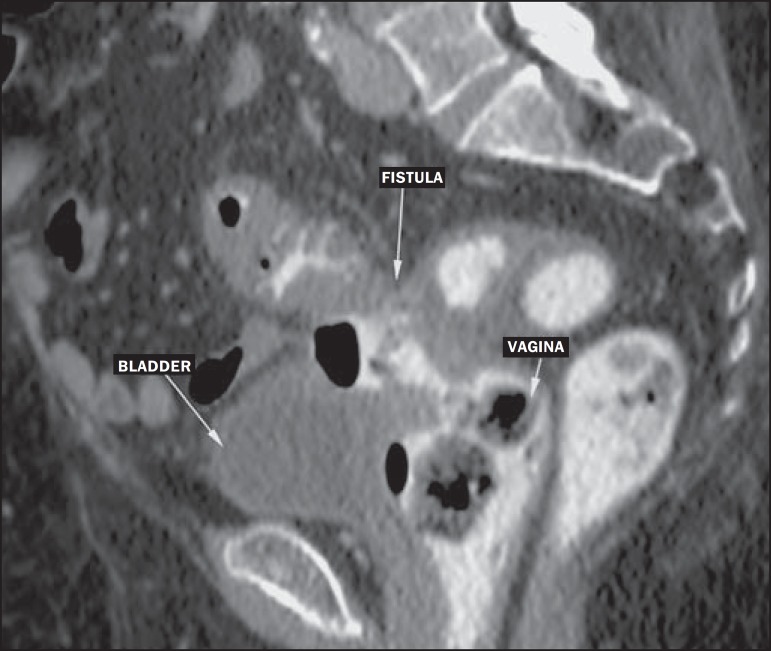
Fistulas to adjacent organs. Rectal contrast-enhanced CT of the abdomen, in the sagittal plane, showing a fistulous pathway between the inflamed colonic segment and the vagina (colovaginal fistula). The diagnostic hypothesis of fistula can be suggested when there is inflammatory tissue or obliteration and increased density of the fat between the colon and the adjacent organs, as well as intraluminal gas (in the bladder, vaginal canal, or other lumen).
– Vascular engorgement (the comb sign), which has a sensitivity of 29% (increasing to 59% if associated with fluid) and a specificity of 100%^(5)^.

## SURGICAL AND TOMOGRAPHIC CLASSIFICATION

In 1978, Hinchey et al. devised a classification system in which acute diverticulitis is categorized into four stages. When the abscess is exclusively pericolonic, it is categorized as stage I, whereas it is categorized as stage II when it extends to the pelvis. When purulent peritonitis occurs, the disease is categorized as stage III. When there is peritoneal dissemination of feces, secondary to a large perforation of the loop, it is categorized as stage IV acute diverticulitis^(6)^.

With the advent of CT in the 1980s, new information could be obtained, which led to various modifications in the initial classification system. Because the Hinchey classification could be applied accurately only in patients who had undergone surgery, it was necessary to create a radiological staging system to assist in the management of acute diverticulitis in patients treated conservatively or with guided punctures^(6)^.

**Figure 7:**
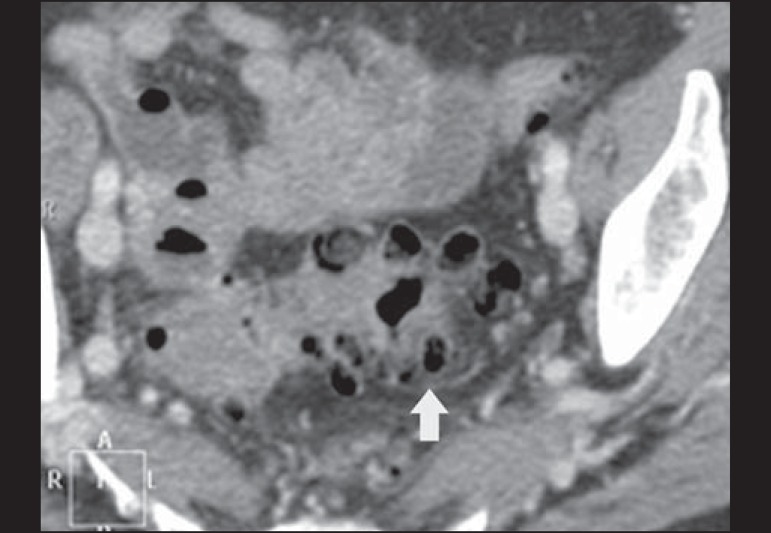
Hinchey stage 0. Intravenous contrast-enhanced abdominal CT, in the axial plane, showing colonic diverticula (arrow), with discrete wall thickening.

**Figure 8:**
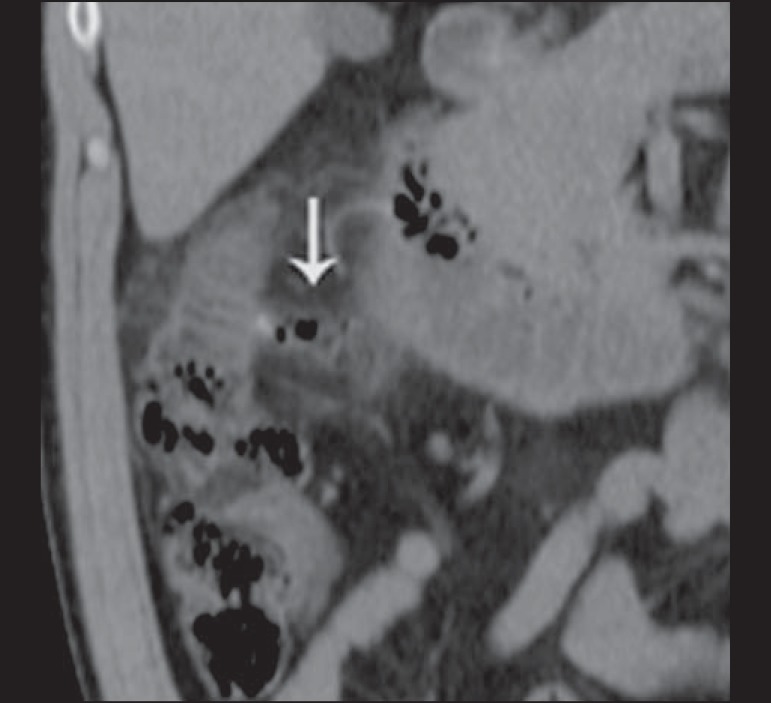
Hinchey stage Ia. Abdominal CT, in the coronal plane, without contrast. Note the wall thickening of the descending colon, accompanied by a perforated diverticulum at the mesenteric border (arrow) and increased density of the adjacent fat, without any fluid collections.

**Figure 9:**
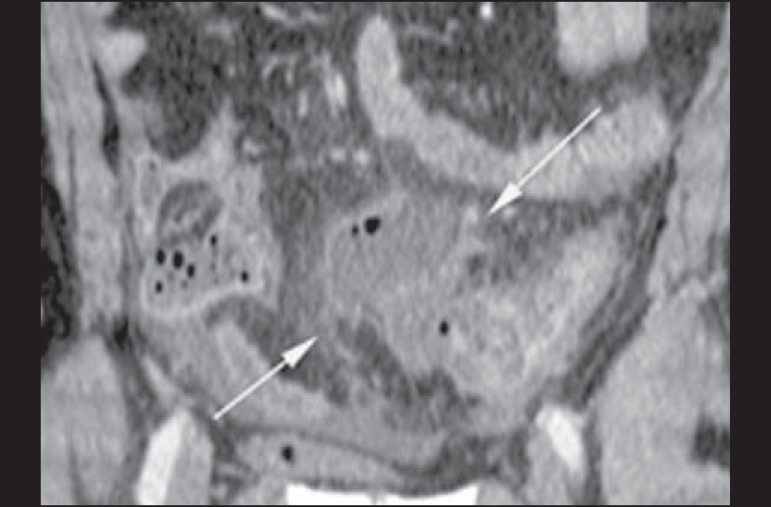
Hinchey stage Ib. Intravenous contrast-enhanced abdominal CT, in the coronal plane, showing wall thickening of the sigmoid, with an adjacent pericolonic abscess (arrows).

**Figure 10:**
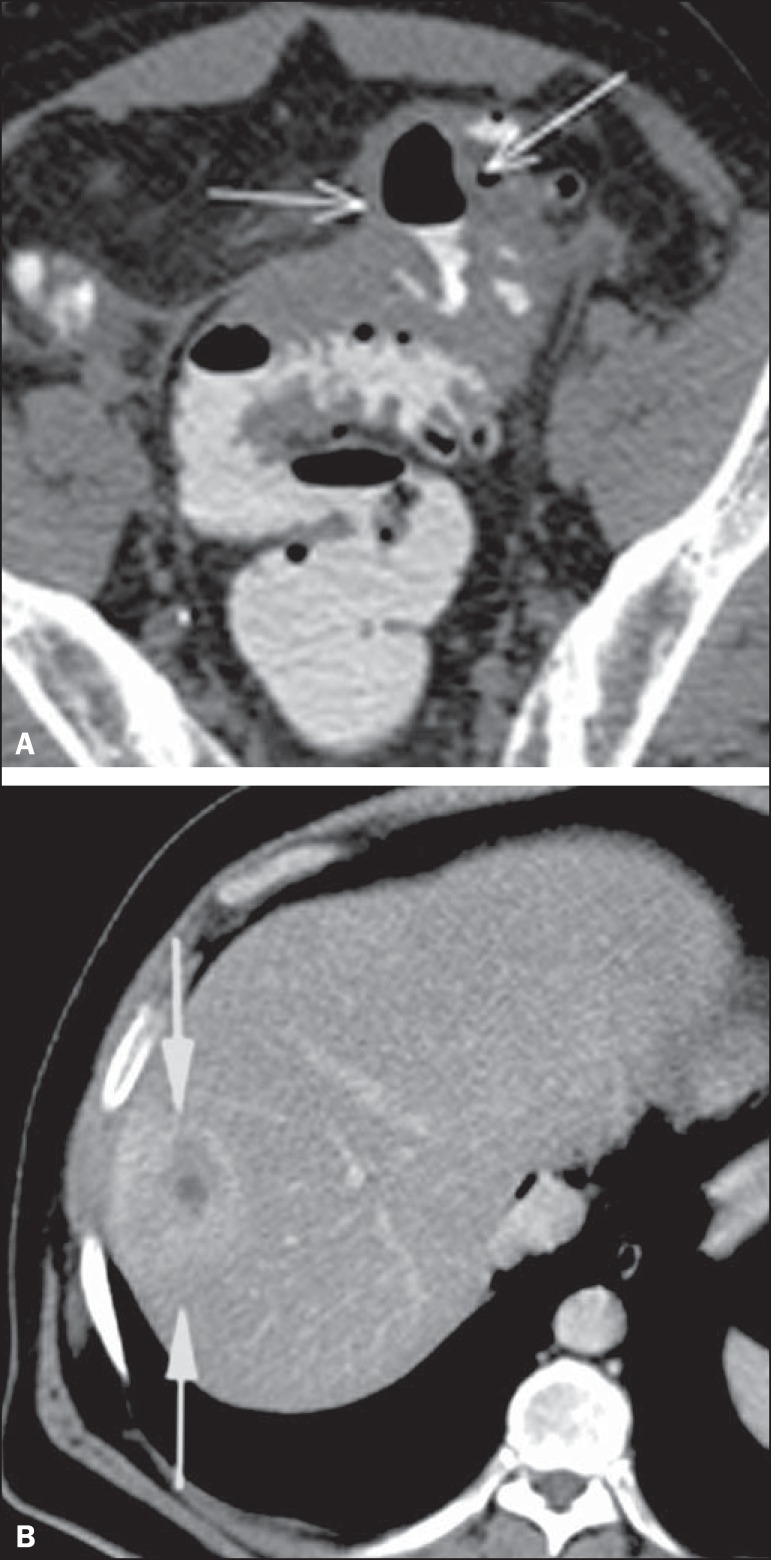
Hinchey stage II. Intravenous and rectal contrast-enhanced axial CT of the abdomen. Note the thickened and finely heterogeneous walls of the sigmoid (arrows in **A**) and the hepatic abscess (arrows in **B**) related to the inflammatory process in the colon.

**Figure 11:**
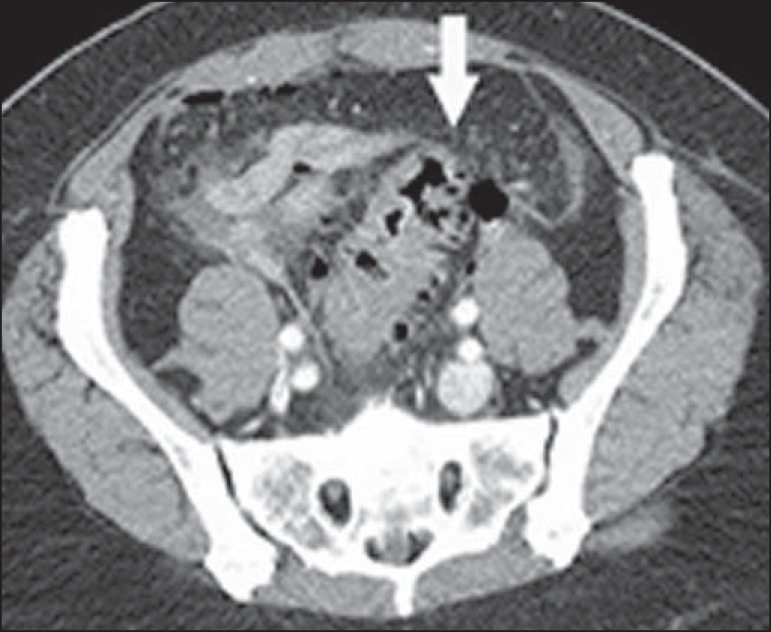
Hinchey stage III. Intravenous and rectal contrast-enhanced axial CT of the abdomen showing diverticulitis with multiple abscesses (arrow) in the inframesocolic region and pneumoperitoneum, together with generalized peritonitis.

**Figure 12:**
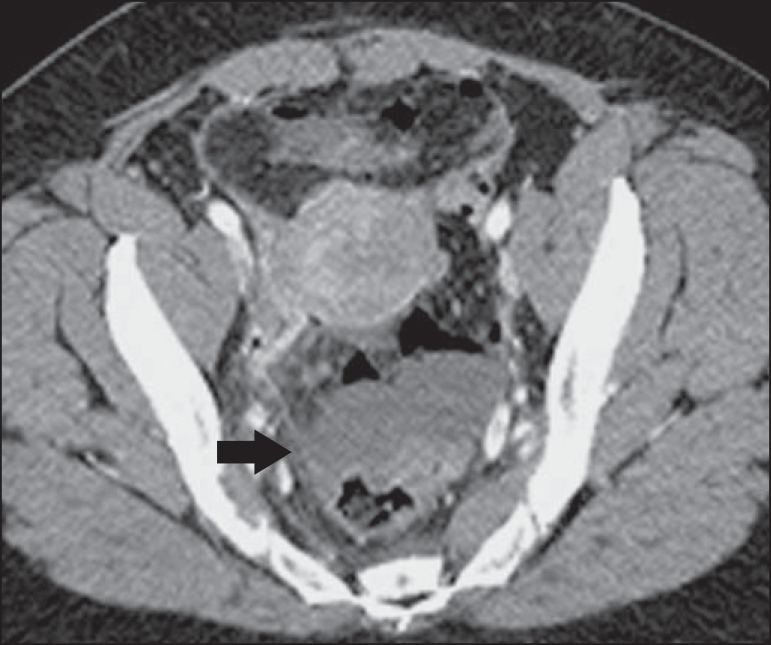
Intravenous contrast-enhanced axial CT of the abdomen showing diverticulitis with perforation and pelvic abscesses with heterogeneous content (arrow) in a patient who subsequently underwent surgery, during which a large amount of pus and feces were found in the peritoneal cavity.

Some surgical guidelines regarding ACD^(6)^ are based on the modifications made to the Hinchey classification by Wasvary et al. and on the CT findings described by Kaiser et al., as shown in Figures 7 to 12.

The most recent classification systems divide ACD into two groups: complicated and uncomplicated. Uncomplicated ACD is characterized only by thickening of the wall of the diverticula, with increased pericolonic fat density. Complicated ACD is divided into stages. In stage 1A, pericolonic air bubbles, with little fluid, can be seen, and there is no abscess. The ACD is classified as stage 1B if the diameter of the abscess is ≤ 4 cm and as stage 2A if it is > 4 cm. In stage 2B there may be distant air (> 5 cm from the inflamed loop); in stages 3 and 4, there is diffuse fluid, without and with distant free air, respectively^(7)^.

Mild and moderate cases of ACD, with only mesenteric fat densification or with small abscesses, can be managed conservatively. Abscesses greater than 5 cm in diameter can be treated with percutaneous drainage or surgery. However, patients presenting with purulent, fecal peritonitis should be treated surgically^(2,7)^.

## COMPLICATIONS

In 5–15% of cases of diverticulitis, fistulous pathways appear after the acute process has resolved. The most common such pathway is a colovesical fistula, which manifests as thickening of the bladder adjacent to thickening of the colonic loop, together with air within the bladder^(3,4)^, as depicted in Figure 6.

The inflammatory process adjacent to the urinary tract can exert a mass effect, causing ureteral obstruction. A similar mechanism can occur in the digestive tract itself, resulting in obstruction that leads to acute abdomen^(4)^.

**Figure 13:**
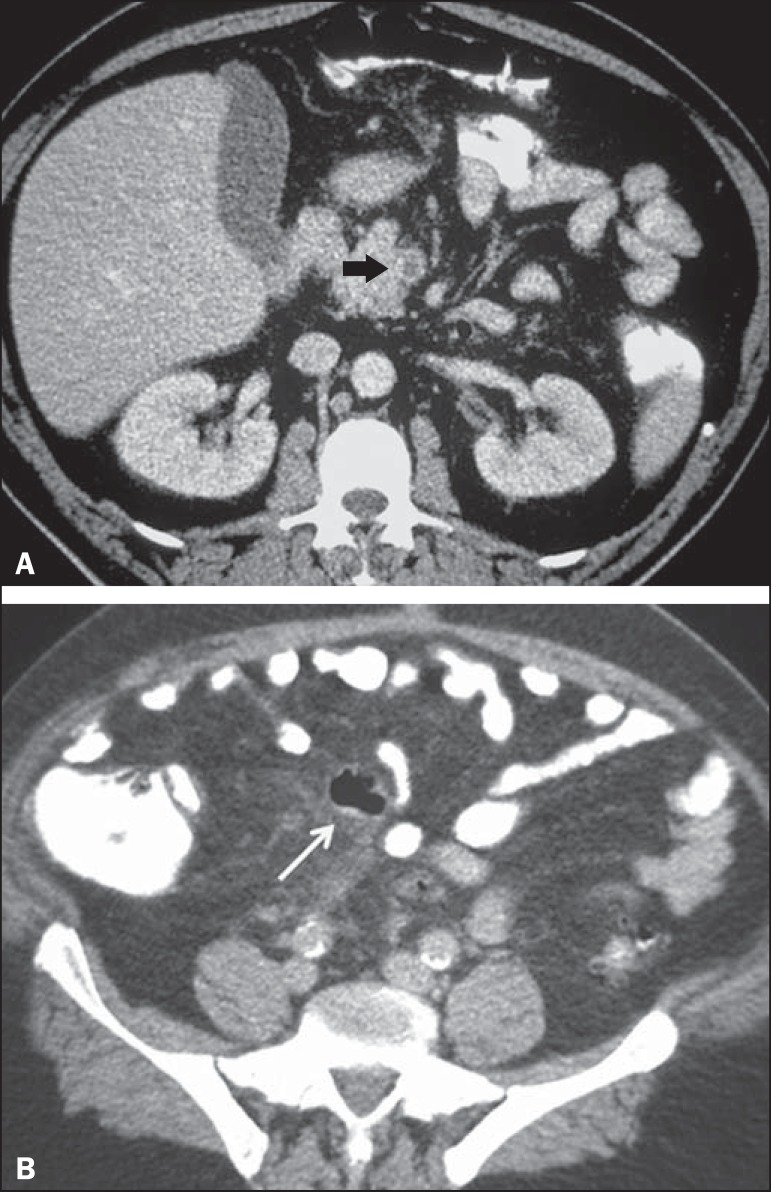
Thrombosis of the portal vein. Intravenous and rectal contrast-enhanced axial CT of the abdomen showing a thrombus within the superior mesenteric vein (arrow in **A**), together with acute perforated and blocked diverticulitis (arrow in **B**), in a patient with diabetes.

Diverticulitis is a common cause of phlebitis or thrombosis of the portal vein, characterized by filling defects or gas within the mesenteric or portal system vessels (Figure 13). Complications include septic embolism, sepsis, venous rupture, and pulmonary thromboembolism^(8)^.

The inflammatory process is disseminated via the mesenteric veins and can thus reach the liver, generating a hepatic abscess. If the abscess is bulky, it causes right-sided diaphragmatic elevation, pleural effusion, and atelectasis^(4,8)^.

## DIFFERENTIAL DIAGNOSES

**Figure 14:**
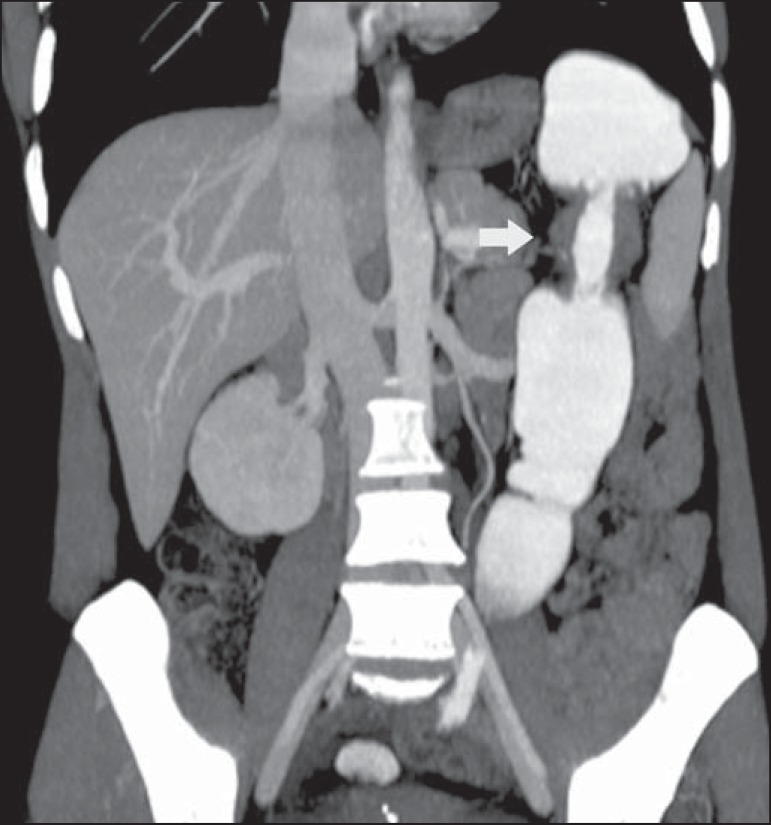
Adenocarcinoma of the colon. Intravenous and rectal contrast-enhanced coronal CT of the abdomen, showing asymmetric wall thickening in the descending colon (arrow), with an abrupt transition to the normal loop (the shoulder sign)

The main differential diagnosis of ACD is adenocarcinoma of the colon, in which the wall thickening is asymmetrical and eccentric, with an abrupt transition to the normal loop, producing the so-called "shoulder sign" (Figure 14). Increased numbers of lymph nodes or lymph node enlargement adjacent to the thickened colon segment also suggest neoplasia, as do signs of distant dissemination of the disease, such as liver and lung metastases^(9)^. When the clinical data are inconclusive, optical colonoscopy is indicated, although it should be performed only after resolution of the acute condition^(10)^.

Infectious enterocolitis can mimic diverticulitis, in terms of the clinical and laboratory findings. In most cases of infectious enterocolitis, the CT scan is normal or shows long, circular, symmetrical segments of intestinal loops with thickened walls, with homogeneous contrast enhancement. Multiple air-fluid levels can be present, as can ascites and inflammation of pericolic fat^(4)^.

## CONCLUSIONS

The main advantages of using CT for the diagnosis of acute diverticulitis are related to the information provided regarding the extent of the extraluminal process. In addition, CT can be used in order to guide interventional procedures.

CT has been considered the exam of choice in the diagnosis of ACD and its complications, allowing the establishment of a treatment strategy that is tailored to the extent and severity of the disease. In most cases, using an examination technique aimed at clinical suspicion, together with systematic evaluation of the examination findings, makes it possible to establish a precise diagnosis with high accuracy.

## References

[1] Tiferes DA, Jayanthi SK, Liguori AAL, D'Ippolito G, Caldana RP (2011). Cólon, reto e apêndice. Gastrointestinal.

[2] Andeweg CS, Mulder IM, Felt-Bersma RJ (2013). Guidelines of diagnostics and treatment of acute left-sided
colonic diverticulitis. Dig Surg.

[3] Sociedade Francesa de Radiologia (2011). Guia de boas práticas médicas em diagnóstico por
imagem.

[4] Horton KM, Corl FM, Fishman EK (2000). CT evaluation of the colon: inflammatory disease. Radiographics.

[5] Kircher MF, Rhea JT, Kihiczak D (2002). Frequency, sensitivity, and specificity of individual signs of
diverticulitis on thin-section helical CT with colonic contrast material:
experience with 312 cases. AJR Am J Roentgenol.

[6] Klarenbeek BR, de Korte N, van der Peet DL (2012). Review of current classifications for diverticular disease and a
translation into clinical practice. Int J Colorectal Dis.

[7] Sartelli M, Moore FA, Ansaloni L (2015). A proposal for a CT driven classification of left colon acute
diverticulitis. World J Emerg Surg.

[8] Perez-Cruet MJ, Grable E, Drapkin MS (1993). Pylephlebitis associated with diverticulitis. South Med J.

[9] Padidar AM, Jeffrey RB, Mindelzun RE (1994). Differentiating sigmoid diverticulitis from carcinoma on CT
scans: mesenteric inflammation suggests diverticulitis. AJR Am J Roentgenol.

[10] Kim MJ, Woo YS, Kim ER (2014). Is colonoscopy necessary after computed tomography diagnosis of
acute diverticulitis. Intest Res.

